# Prevalence of and Risk Factors for Lipoatrophy in Patients with HIV Infection in Nigeria

**DOI:** 10.1155/2015/402638

**Published:** 2015-03-02

**Authors:** Sandra Omozehio Iwuala, Olufunmilayo A. Lesi, Olufemi Adetola Fasanmade, Anas A. Sabir, Michael Adeyemi Olamoyegun, Charles C. Okany

**Affiliations:** ^1^Department of Medicine, College of Medicine University of Lagos, PMB 12003, Lagos, Nigeria; ^2^Department of Medicine, Usmanu Danfodiyo University Teaching Hospital, PMB 2370, Sokoto, Nigeria; ^3^Department of Medicine, LAUTECH Teaching Hospital and College of Health Sciences, Ladoke Akintola University of Technology, PMB 4007, Ogbomoso, Oyo State, Nigeria; ^4^Department of Haematology and Blood Transfusion, Lagos University Teaching Hospital, PMB 12003, Lagos, Nigeria

## Abstract

*Background*. Although the association between lipoatrophy and highly active antiretroviral therapy (HAART) is well known, other nondrug factors may be associated with lipoatrophy in people living with HIV/AIDS (PLWHA). There are no reports of lipoatrophy from Nigeria, a country with the second largest number of PLWHA. We aimed to determine the prevalence, characteristics, and factors associated with lipoatrophy in a cohort of patients attending the HIV clinic in Lagos University Teaching Hospital, Nigeria. *Methods*. Two hundred and eighty-eight patients with HIV infection were recruited for the study. The study protocol involved administration of a questionnaire, targeted physical examination (including anthropometric indices and skin fold thickness), and bioelectrical impedance analysis measurements. Lipoatrophy was defined clinically. *Results*. Lipoatrophy was present in 75 (26.0%) persons. It was associated with lower body circumferences, skin fold thicknesses, and lower % body fat with preservation of skeletal muscle mass (all *P* < 0.05). Male gender and HAART use were the factors associated with lipoatrophy on multivariate analysis (*P* < 0.05). *Conclusion*. Lipoatrophy is frequently encountered in patients with HIV infection in Nigeria, with HAART use conferring an added factor in its development. There is need for more physician and patient awareness of this condition.

## 1. Introduction

The effective use of highly active antiretroviral therapy (HAART) since 1996 has led to a decline in the morbidity and mortality associated with the HIV infection [1, 2]. However, a myriad of adverse effects associated with HAART including fat redistribution or lipodystrophy was increasingly being noted [3–5]. Lipodystrophy can present with lipoatrophy (peripheral fat wasting), lipohypertrophy (central fat accumulation), or a combination of both [5, 6]. While lipohypertrophy can present independently of HIV infection and HAART, lipoatrophy has been linked with HAART especially with nucleoside reverse transcriptase inhibitors (NRTI), which often form the backbone of HAART in resource-poor settings [5, 7]. The reported prevalence of lipoatrophy varies widely due to the difference in tests used in its assessment, differences in the population studied, the duration of follow-up, and HAART regimens used in the various studies. Studies from different parts of the world report prevalence ranging from 13.3 to 52.9% [8–11].

Lipoatrophy affects cosmetic appearance, thus contributing to stigma and potentially influencing long-term adherence to HAART with attendant consequences [12, 13]. It is also damaging to self-image and can reduce quality of life of affected persons [14]. As part of the lipodystrophy syndrome, it may be also associated with metabolic complications such as dyslipidemia, glucose intolerance, and insulin resistance, which contribute to HIV related morbidity and mortality through increased cardiovascular and cerebrovascular disease risk [15, 16].

In recent years there has been more widespread access to antiretroviral therapy and PLWHA even in Africa where the greatest burden of the infection is expected to live longer. A few studies have been carried out on lipodystrophy in Africa [10, 17, 18]. However, there are no published studies on lipoatrophy in Nigeria, which bears the second largest burden of HIV infection in Africa after South Africa. The current study aimed to study the prevalence and factors associated with lipoatrophy in HAART experienced patients attending the HIV clinic in Lagos University Teaching Hospital, Lagos, Nigeria.

## 2. Subjects and Methods

### 2.1. Setting and Design

This cross-sectional study was carried out among patients attending the HIV outpatient clinic of Lagos University Teaching Hospital (LUTH) where free health care of over 6,000 persons living with HIV infection including provision of HAART takes place. Approval for the study was obtained from the Health Research and Ethics Committee of LUTH. Written informed consent was obtained from the subjects.

### 2.2. Subjects

Ambulant consecutively consenting HIV positive patients on HAART meeting the inclusion criteria were recruited into the study. Patients were included in the study if they were confirmed to have HIV infection, were aged between 15 and 70 years, were not on drugs affecting carbohydrate and fat metabolism or inducing lipodystrophy for example statins, corticosteroids, metformin, and thiazolidinediones, and were not known patients with diabetes mellitus or tuberculosis. Patients on HAART were included if they had been on HAART for 6 months and above. HIV seropositive patients who were moribund, acutely ill, pregnant, or lactating were excluded from the study. HAART naïve patients meeting the inclusion criteria were also recruited.

### 2.3. Study Protocol

The study protocol involved the administration of a questionnaire, physical examination, and laboratory evaluation including the CD4 count and HIV RNA viral load.

Information regarding the patients' demographic characteristics, lifestyle, personal and family disease history, and drug history was obtained. Patients' reports of body fat changes were also obtained. In the questionnaire, patients were asked about changes in the size of their clothing and body measures. The possible responses were “yes,” “no,” or do not know. The degree of fat redistribution in six regions (face, arms, legs, and buttocks) was graded using the HIV outpatient study (HOPS) scale as follows: absent (score of 0), mild (no change in clothing size/noticeable on close inspection; score of 1), moderate (clothing has become loose/readily noticeable by patient or physician; score of 2), or severe (change in clothing size/readily noticeable to a casual observer; score of 3) [19]. A targeted physical examination evaluating lipodystrophy was done for all study participants by a single physician. This was followed by measurement of the following anthropometric indices: height, weight, waist, hip and midupper arm circumferences, skin fold thicknesses, and blood pressure measurement by standard methods. Body composition was assessed with bioimpedance analysis (BIA) using the Omron body composition monitor with scale (HBF-500).

The CD4 cell count was done by the use of Partec flow cytometer and CD4 Easy Count kit made by Partec Healthcare. HIV viral load was quantified by polymerase chain reaction (PCR) using AMPICLOR HIV-1 Monitor Test (Roche). The CD4 and viral load values were retrieved from the patients' clinical notes.

### 2.4. Main Outcome Measure

The main outcome measure for this study was lipoatrophy, which was defined as self-report of loss of fat from the face, arms, legs, and buttocks supported by targeted physical examination or fat wasting from the face, arms, legs, or buttocks detected on physical examination [20, 21].

### 2.5. Statistical Analysis

The data was analysed using epi-info version 3.4.3 statistical software. Continuous variables were expressed as means and standard deviation. Categorical variables were expressed as frequencies with accompanying percentages in paracentesis. Differences between groups were compared using the chi-square and Student's *t*-test for categorical and continuous variables, respectively. Multivariate logistic regression analysis was used to determine the factors associated with lipoatrophy. Odds ratio and the corresponding 95% confidence intervals (CI) were presented. A *P* < 0.05 was accepted as significant.

## 3. Results

Three hundred (300) patients were recruited for the study; however, analysis was based on 288 persons of which 145 were HAART experienced and 143 were HAART naive. Five HAART experienced and seven HAART naive patients were excluded for various reasons such as incomplete data and lack of recent CD4 cell count.

### 3.1. Sociodemographic and HIV Related Characteristics of the Study Participants

The sociodemographic and HIV characteristics of the study participants are shown in Table 1. The mean (SD) age was 39.6 ± 8.9 years. There were 172 (59.7%) females and 116 (40.3%) males. Majority (78.8%) of the patients had completed secondary school. The median (IQR) of the known duration of HIV infection was 22.0 (8.0–42.0) months. The median (IQR) CD4 count was 350 (249–515) cells/mm^3^. The highest frequency of CD4 category was 201–500 cells/mm^3^, with 170 (59.0%) persons in that category.

One hundred and forty-five patients were on HAART and 143 were HAART naïve. There was no significant difference in the age or gender distribution between the patients on HAART and those who were HAART naïve. The median duration of HAART was 29.0 (16.0–40.0) months.

All the patients on HAART (145) had nucleoside and/or nucleotide analogs in their regimen. While 62/145 (42.8%) were on a zidovudine containing regimen, 48/145 (33.1%) were on stavudine containing regimen, and 35/145 (24.1%) were on tenofovir containing regimen. These three drugs were mutually exclusive in the study population and so HAART regimen was referred to as stavudine based, zidovudine based, or tenofovir based. One hundred and thirty-five patients (93.1%) were on nonnucleoside reverse transcriptase inhibitors (NNRTI), comprised of 100 on nevirapine and 35 on efavirenz. Only 9/145 (6.2%) patients were on protease inhibitors; one patient was on 3 NRTIs.

### 3.2. Anthropometric Indices and Skin Fold Thicknesses in the Study Participants

The anthropometric indices, skin fold thicknesses, and body composition using the consumer bioelectrical impedance analysis (BIA) device of the patients are shown in Table 2.

### 3.3. Frequency of Lipoatrophy in the Study Participants

The overall frequency of lipoatrophy in the study population was 75 (26.0%). This was made up of 48 (33.1%) HAART experienced patients and 27 (18.9%) HAART naive patients. The difference in frequency was statistically significant (OR = 2.13, 95% CI 1.19–3.80, and *P* = 0.009).

### 3.4. Self-Report of Body Fat Changes

Of the 75 patients diagnosed with lipoatrophy in the study population, only 22 (29.3%) at questioning reported body fat changes.

### 3.5. Pattern and Severity of Lipoatrophy in the Study Participants

The face was the most frequent site affected by lipoatrophy, being involved in 52 (69.3%) out of 75 persons with lipoatrophy. This was followed by upper limb, buttock, and lower limb involvement as shown in Table 3. Lipoatrophy was mostly mild in severity. Forty-six out of 75 persons had HOPS grade 1 lipoatrophy.

### 3.6. Demographic, Anthropometric, and BIA Characteristics of Lipoatrophy in the Study Participants

The mean age of persons with lipoatrophy was significantly higher than those without lipoatrophy. Males had a significantly higher prevalence of lipoatrophy compared to females.

Among the anthropometric indices studied, subjects with lipoatrophy had a significantly lower BMI, WC, HC, MUAC, triceps, biceps, suprailiac, subscapular, sum of skin fold thickness, and % of body fat, compared to the HIV patients without lipoatrophy. These are shown in Table 4.

### 3.7. HIV Related Characteristics of Lipoatrophy in the Study Participants

There was no significant difference in the known duration of HIV infection and viral load (copies/mL) in the HIV positive patients with or without lipoatrophy. However, HIV patients with lipoatrophy were less likely to have a CD4 count greater than 500 mm^3^ and to be on a tenofovir based HAART regimen. They also had a lower median CD4 count compared to those without lipoatrophy. The comparison of HIV related characteristic of the study population with or without lipoatrophy is shown in Table 4.

### 3.8. Factors Associated with Lipoatrophy

The association of lipoatrophy with patient, treatment, and disease related factors was assessed using logistic regression.  Table 5 summarizes the predictive value of several variables in the development of lipoatrophy in HIV positive patients using regression analysis. After correcting for confounding variables, only 2 of the independent variables made significant contribution to the regression model: male gender (OR = 4.34, 95% CI 2.29–8.23, and *P* < 0.001) and HAART use (OR = 2.31, 95% CI 1.12–4.73, and *P* = 0.03).

## 4. Discussion

### 4.1. Prevalence of Lipoatrophy

These study findings demonstrate that lipoatrophy is a frequent consequence of HIV infection and/or its treatment. Amongst the 288 HIV study participants, 75 (26.0%) had lipoatrophy. The prevalence of lipoatrophy in patients on HAART in this study was 33.1%. Lower prevalence of 13.1% to 29.3% of lipoatrophy amongst patients on HAART in developing countries has been reported [10, 17, 20]. In the developed world, some workers reported lower prevalence of 14% to 16% [19, 22]. Others found higher values ranging 38.3% to 52.9% compared to that obtained in this study [8, 9, 11, 23].

The differences in these prevalence rates may be explained by the following reasons: differences in duration and type of HAART use, definition/assessment of lipoatrophy, study design, and patient factors. For instance, the higher prevalence of lipoatrophy amongst patients on HAART found in this study compared to that found by van Griensven et al. (Rwanda) [17] and Pujari et al. (India) [20] could be attributed to the longer duration of HAART in this study (29 versus 16 and 18 months, resp.). The pathogenic mechanisms inducing lipoatrophy would be present for long with longer duration of therapy. Hansen et al. in their study of HIV positive men, who had been on HAART for 13.3 years, reported a 52.9% prevalence of lipoatrophy [11].

In this study, lipoatrophy was defined clinically and was weighted on physician assessment of lipoatrophy as has been done by other workers [20, 21]. There was a low prevalence of self-report on questioning for body fat loss in the study population with lipoatrophy. This has been reported in other studies done in developing countries [20, 21]. This may be partially due to patients' lack of awareness of the morphological effects of HAART. Also, overall weight gain and well-being after commencement of HAART may have precluded the observation of fat loss from certain body sites. Even in the HOPS study (USA), physician assessment of the severity of lipoatrophy prevailed when there were differences between physician and patients assessment [19]. Though an objective case definition for diagnosing lipodystrophy syndrome in general has been suggested, its reliability in the absence of tools like DEXA is low [24]. DEXA was not available at this study site and so could not be employed in this study for regional body composition.

The finding of 18.9% of lipoatrophy in HAART naive patients demonstrates that lipoatrophy is not restricted to HIV patients on drugs, but that HAART use confers an increased risk to its development. Lichtenstein et al. identified other diagnoses that could have caused lipoatrophy in their cohort of PLWHA and influenced their results [25]. They were* Mycobacterium avium* infection, renal failure, pneumonia, depression, and reactive airway disease. These were, however, not sought for in this study though known patients with tuberculosiswere excluded from this study.

### 4.2. Factors Associated with Lipoatrophy

The factors associated with lipoatrophy assessed for in this study include host, treatment, and disease factors. Male gender and HAART were the factors associated with lipoatrophy even after adjustment for confounding variables in this study. There are conflicting reports about the association of gender with lipoatrophy. While some studies found lipoatrophy to be more frequent in males [9, 26], others have found females to be more at risk [17] and some did not find gender [27] to be associated with lipoatrophy. Males physiologically have less body fat than females and so may be more prone to the pathologic mechanisms inducing lipoatrophy.

Being on HAART in this study was a significant risk factor associated with lipoatrophy even after adjustment for confounding variables. Much evidence exists to demonstrate that NRTI associated fat tissue mitochondrial toxicity plays a dominant role in the pathogenesis of lipoatrophy [28–30]. Mitochondria produce energy by generating adenosine triphosphate (ATP) through the oxidative phosphorylation of glucose and fatty acids. Mitochondria, unlike other cellular organelles, contain their own extrachromosomal genome and mitochondrial DNA (mtDNA) replicates independently of nuclear DNA using mtDNA polymerase gamma which NRTIs selectively inhibit, leading to mitochondrial DNA depletion. Some authors have alsoproposed that NRTI associated mitochondrial toxicity occurs when mtDNA falls below a critical level insufficient to meet energy requirements. The resultant mitochondrial dysfunction impairs the function of adipocytes via increased levels of reactive oxygen species and reduced ATP production [31]. In our study, all the patients on HAART were on NRTI based regimens. There was a statistically significant difference in the HAART regimen of patients on treatment who had lipoatrophy. Current or past stavudine use, amongst the NRTIs, has been frequently implicated in the development of lipoatrophy [17, 32–36].

The pathologic changes observed in the adipose tissue of patients with lipoatrophy include increased apoptosis, adipocyte pleomorphism, loss of tissue architecture, increased fibrosis, macrophage infiltration, and increased levels of inflammatory cytokines [30, 37].

Other disease related factors (CD4 count and viral load) were not also significantly associated with lipoatrophy on multivariate analysis. Basal CD4 counts as well as nadir CD4 count are the disease related factors that have been found to be associated with lipoatrophy [17, 27, 38]. These data were not available in this study. This area is a possible thrust for further research in Nigeria.

Our study is not without limitations. The cross-sectional study design is unable to prove causality of lipoatrophy among our cohorts. The absence of DEXA at the study site which precluded the use of an objective case definition of lipoatrophy or magnetic resonance imaging (MRI) and computer tomography (CT) for regional body fat distribution is also a study limitation. However, some objective methods of assessing body fat such as anthropometric indices and % of body fat using BIA the anthropometric indices were utilized in this study. A good correlation (*r* ^2^ = 0.92–0.96, *P* < 0.001) between measures of body composition (fat mass and skeletal muscle mass) determined by DEXA or MRI has been reported [39]. Larger studies incorporating the prediction of lipoatrophy with the use of cheap and readily available methods of regional body fat distribution such as skin fold calipers among PLWHA, especially those on treatment in resource poor settings, are a thrust for further research.

In summary, this study has demonstrated that lipoatrophy is prevalent in HIV infected individuals, especially males and those on HAART. Regular monitoring by physicians and increased patients awareness are necessary to identify it and reduce its potential impact on patients with HIV infection.

## Figures and Tables

**Table 1:** Sociodemographic and HIV related characteristics of the study participants.

Variable	*N* = 288 (%)
Mean age (years)	39.6 ± 8.9
Gender distribution	
Female *n*, %	172 (59.7)
Male *n*, %	116 (40.3)
Known duration of HIV (months)	
<12	92 (31.9)
12–36	114 (39.6)
37–60	46 (16.0)
≥61	36 (12.5)
Median (IQR)	22 (8.0–42.0)
CD4 count (cells/mm^3^)	
<200	82 (28.5)
201–500	170 (59.0)
≥500	36 (12.5)
Viral load (copies/mL)	
<200	143 (49.7)
≥200	145 (50.3)
On HAART	
Yes	145 (50.3)
No	143 (49.7)
Drug regimen	
Stavudine based	48/145 (33.1)
Zidovudine based	62/145 (42.8)
Tenofovir based	35/145 (24.1)
Median (IQR) duration of HAART (months)	29.0 (16.0–40.0)

HAART: highly active antiretroviral therapy.

**Table 2:** Anthropometric indices in the study participants.

Variable	*n* = 288
Body mass index (kg/m^2^)	25.1 ± 4.6
Waist circumference, male (cm)	88.2 ± 11.4
Waist circumference, female (cm)	84.5 ± 11.3
Hip circumference, male (cm)	99.0 ± 9.1
Hip circumference, female (cm)	100.4 ± 10.6
Waist-to-hip ratio (male)	0.89 ± 0.07
Waist-to-hip ratio (female)	0.84 ± 0.06
Neck circumference (cm)	34.5 ± 3.4
Midupper arm circumference (cm)	29.8 ± 4.3
Median (IQR) skin fold thickness (mm)	
Triceps	11.2 (6.0–19.0)
Biceps	5.2 (3.3–9.0)
Suprailiac	7.0 (4.0–10.8)
Subscapular	14.3 (10.0–23.0)
Sum skin fold thickness	39.0 (25.7–64.0)
Median body composition measurement	
% of body fat	28.9 (19.4–37.4)
% of skeletal muscle mass	20.4 (16.6–35.6)

^*^Statistically significant; all values are mean (SD) unless stated otherwise.

**Table 3:** Prevalence, self-report, and pattern of lipoatrophy in the study participants.

Variable	Frequency	%
Prevalence of lipoatrophy	75/288	26.0
Gender distribution of lipoatrophy		
Male	48	64.0
Female	27	36.0
Self-report of body fat loss	22/75	29.3
Body region affected^†^		
Face	52/75	69.3
Upper limbs	30/75	40.0
Lower limbs	22/75	29.3
Buttocks	25/75	33.3
Number of body regions affected		
1	38/75	50.7
2	21/75	28.0
3	11/75	14.7
4	5/75	6.7
Worst severity score^‡^		
1	46/75	61.3
2	27/75	36.0
** **3	2/75	2.7

^†^Values are overlapping, and ^‡^HOPS grade: grade 1 (mild), grade 2 (moderate), and grade 3 (severe).

**Table 4:** Demographic, anthropometric, and body composition of lipoatrophy in the study participants.

Characteristic	Lipoatrophy *n* = 75	No lipoatrophy *n* = 213	*P* value
Demographic			
Age (years)	41.7 ± 8.8	38.9 ± 8.6	0.02^*^
Gender			
Male (*n*, %)	48 (41.4%)	68 (58.6%)	0.0001^*^
Female (*n*, %)	27 (15.7%)	145 (84.3%)	
Anthropometric			
BMI, male (kg/m^2^)	23.1 ± 3.3	26.5 ± 3.8	0.0001^*^
BMI, female (kg/m^2^)	21.2 ± 2.1	25.8 ± 5.0	0.0001^*^
WC, male (cm)	82.1 ± 9.3	92.6 ± 10.7	0.0001^*^
WC, female (cm)	77.0 ± 6.2	85.9 ± 11.5	0.0001
HC, male (cm)	93.2 ± 6.3	103.2 ± 8.5	0.0001^*^
HC, female (cm)	90.8 ± 5.1	102.2 ± 10.4	0.0001^*^
WHR, male	0.88 ± 0.08	0.90 ± 0.06	0.49
WHR, female	0.85 ± 0.06	0.84 ± 0.06	0.25
Median triceps (mm), male	5.0 (3.3–7.2)	9.3 (5.0–12.7)	0.0001^*^
Median (IQR) triceps, female (mm)	8.7 (6.0–11.0)	18.0 (11.0–25.0)	0.0001^*^
Median SST, male (mm)	23.7 (17.2–30.3)	34.7 (25.0–48.7)	0.0001^*^
Median SST, female (mm)	27.0 (23.3–36.7)	51.0 (38.7–119.7)	0.0001^*^
Median % of body fat, male	14.1 (13.0–16.7)	23.6 (17.9–28.0)	0.0001^*^
Median % of body fat, female	25.1 (19.9–27.7)	37.2 (31.1–55.5)	0.0001^*^
Median % of skeletal muscle mass, male	41.0 (38.9–42.6)	35.7 (33.0–38.4)	0.0001^*^
Median % skeletal muscle mass, female	31.5 (24.9–35.1)	26.4 (24.1–28.8)	0.0001^*^
Median (IQR) known duration of HIV infection (months)	30.0 (8.0–49.0)	20 (7.5–30.0)	0.06
CD4 count (cells/mm^3^)			
>500	13 (15.9)	69 (84.1)	0.04^*^
200–500	50 (29.4)	120 (70.6)	
<200	12 (33.3)	24 (66.7)	
Median (IQR)	309 (242–409)	356 (253–539)	0.03^*^
Viral load (copies/mL)			
<200	45 (31.5)	98 (68.5)	0.05
≥200	30 (20.7)	115 (79.3)	
Total number on HAART	48 (33.1%)	97 (67.9%)	0.01^*^
Median (IQR) duration of HAART (months)	32.0 (15.5–49.0)	26.0 (16.0–38.0)	0.29
HAART regimen			
Stavudine based	22 (45.8%)	26 (54.2%)	0.04^*^
Zidovudine based	19 (30.6%)	43 (69.4%)	
Tenofovir based	7 (20.0%)	28 (80.0%)	

^*^Statistically significant; BMI: body mass index, WC: waist circumference, HC: hip circumference, and WHR: waist-to-hip ratio. All values are mean ± SD unless stated.

**Table 5:** Logistic regression of factors predicting the development of lipoatrophy in the patients with HIV infection.

Variable	Odds ratio	95% CI	*P* value
Host factor			
Age	1.03	0.99–1.07	0.15
Gender (male/female)	4.34	2.29–8.23	<0.001^*^
Treatment factor			
HAART use (yes/no)	2.31	1.12–4.73	0.03^*^
Disease factor			
CD4 count (mm^3^)	1.00	1.00-1.00	0.09
Viral load (copies/mL) <200/>200	1.76	1.03–3.00	0.46
Known duration of HIV infection	1.03	0.53–2.00	0.94

CI: confidence interval; ^*^statistically significant.
